# Docking into *Mycobacterium tuberculosis* Thioredoxin Reductase Protein Yields Pyrazolone Lead Molecules for Methicillin-Resistant *Staphylococcus aureus*

**DOI:** 10.3390/antibiotics6010004

**Published:** 2017-01-28

**Authors:** Noreena L. Sweeney, Lauren Lipker, Alicia M. Hanson, Chris J. Bohl, Katie E. Engel, Kelsey S. Kalous, Mary E. Stemper, Daniel S. Sem, William R. Schwan

**Affiliations:** 1 Department of Pharmaceutical Sciences, Concordia University Wisconsin, 12800 N Lake Shore Dr, Mequon, WI 53097, USA; noreena.sweeney@cuw.edu (N.L.S.); alicia.schultz@cuw.edu (A.M.H.); chris.bohl@cuw.edu (C.J.B.); Daniel.Sem@cuw.edu (D.S.S.); 2 Department of Microbiology, University of Wisconsin-La Crosse, La Crosse, WI 54601, USA; lipker.laur@uwlax.edu (L.L.); katieengel92@gmail.com (K.E.E.); 3 Department of Biochemistry, Medical College of Wisconsin, Wauwatosa, WI 53226, USA; kekalous@mcw.edu; 4 Marshfield Clinic Research Foundation, Marshfield, WI 54449, USA; stemper.mary@marshfieldclinic.org

**Keywords:** thioredoxin reductase, antibiotic target, molecular docking, enzyme inhibitor, MRSA, pyrazolones

## Abstract

The thioredoxin/thioredoxin reductase system (Trx/TrxR) is an attractive drug target because of its involvement in a number of important physiological processes, from DNA synthesis to regulating signal transduction. This study describes the finding of pyrazolone compounds that are active against *Staphylococcus aureus*. Initially, the project was focused on discovering small molecules that may have antibacterial properties targeting the *Mycobacterium tuberculosis* thioredoxin reductase. This led to the discovery of a pyrazolone scaffold-containing compound series that showed bactericidal capability against *S. aureus* strains, including drug-resistant clinical isolates. The findings support continued development of the pyrazolone compounds as potential anti-*S. aureus* antibiotics.

## 1. Introduction

Thioredoxin reductases (TrxRs) belong to a family of flavoproteins with redox-active cysteine residues that link the pyridine nucleotides with sulfur-containing substrates (thioredoxin proteins) [1]. Each monomer in this homodimeric protein includes an NADPH binding site, a FAD prosthetic group, and an active site containing a redox-active disulfide [2]. TrxRs get their name because they catalyze the reduction of oxidized small protein thioredoxin (Trx), which belongs to a group of small (10–12 kDa) redox-active proteins with a conserved Trp-Cys-Gly-Pro-Cys-Lys catalytic motif [3]. Reduction of the substrate disulfide occurs when electrons are transferred from NADPH via FAD to the sulfur-containing active site of TrxR.

The Trx system—collectively comprised of Trx, TrxR and NADPH—plays an essential role in various physiological processes, including regulating cellular viability and function, redox homeostasis, reducing nucleotides to deoxyribonucleotides, and helping to detoxify the body from oxidants and free radicals [4]. Therefore, it is important as a clinical goal to target the inhibition of TrxR in pathogenic microbes, since it is involved in the above-mentioned physiological/pathological processes.

One particular infectious disease that can be attacked by targeting the Trx system is tuberculosis (TB), untreated infections of which resulted in 9.6 million cases of active TB and 1.5 million deaths in 2014 [5]. *Mycobacterium tuberculosis* (*M. tuberculosis*, the causative agent of TB) requires high levels of oxygen, and is primarily a pathogen of the mammalian respiratory lungs [6]. The exact mechanism by which it resists the oxidative killing of immune cells has yet to be fully elucidated; but it is clear that the proteins involved in this defense are attractive drug targets [7].

Another bacterial species that could be targeted by a drug that is specific for the Trx system is *Staphylococcus aureus* (*S. aureus*)*.* This Gram-positive bacteria is a significant cause of skin and soft tissue infections as well as life-threatening diseases such as pneumonia, endocarditis, and bloodstream infections [8]. Within the genome of *S. aureus*, there is a single TrxR gene (*trxB*), and the Trx system is essential for its growth [9]. Thus, the Trx system is an attractive drug target for multiple infectious diseases.

In recent years, numerous groups have demonstrated the feasibility of targeting the Trx system in bacteria as a unique antibacterial drug discovery approach. Some of the compounds found to inhibit the Trx system have included epigallocatechin-3-gallate [10], ebselen [11,12,13], auranofin [14,15], and gold(I)-alkynyl chromones [16]. The bacterial species that have been targeted by these compounds have included *Bacillus subtilis* (*B. subtilis*), *Escherichia coli* (*E. coli*), *Enterococcus faecalis* (*E. faecalis*), *Enterococcus faecium* (*E. faecium*), *Helicobacter pylori* (*H. pylori*), *M. tuberculosis*, and *S. aureus*. Clearly, the Trx system is a viable target for the discovery of potent antibacterial drugs.

Both high-throughput screening and virtual screening have been successfully used to identify new leads in drug discovery [17]. The program AUTODOCK has been effectively used in various protein systems for virtual screening [18,19]. In the present study, we initially attempted to identify *M. tuberculosis* TrxR inhibitors by virtual screening of compound libraries, followed by activity assay and in silico structural characterization. After an initial drug lead was identified, derivatives of the lead molecule were found by doing a similar scaffold search from a compound database. Several lead compounds—identified in silico—were screened against six bacterial species that included two mycobacterial species as well as *S. aureus* and *Enterococcus faecalis* (*E. faecalis*). Although none of the lead compounds showed minimum inhibitory concentration (MIC) activity against the two mycobacterial species that were tested, some of them did display antibacterial activity against drug-resistant *S. aureus*.

## 2. Results and Discussion

### 2.1. Initial Virtual Screening and Bacterial Assays

**Figure 1:**
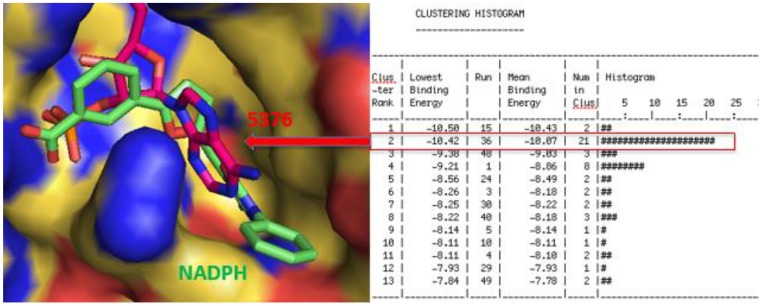
Docking energy and binding pose for compound 5376 in its lowest energy and highest populated cluster (Run 36, Cluster 2).

The project started with docking of compounds from the Center for Structure-based Drug Design and Development (CSD^3^) screening collection against a crystal structure of *M. tuberculosis* TrxR (PDB accession code 2A87) [7]. Nine compounds with the best-predicted binding energy (Figure 1) were then tested against two *Mycobacterium* species: *Mycobacterium marinum* and *Mycobacterium smegmatis*. Surprisingly, the MIC results did not support the predicted binding energies from the molecular docking (Table 1). We hypothesize that this is probably due to the lack of cell wall penetration, due to the thick mycolic acid lipid layer surrounding the mycobacterial cells.

The same nine compounds were then used to run MIC assays against a panel of four other bacterial species (Table 1). Interestingly, some of the compounds showed activity against both *S. aureus* and *E. faecalis*, with compound CSD^3^ 5376 (therein called 5376) having the best MIC of 8 µg/mL against *S. aureus* bacteria. Compound 1882 had activity against both *S. aureus* and *E. faecalis*, although the MIC values demonstrated only a modicum of efficacy against both species. (MIC values of 32 μg/mL for each species). Additional work could be done in the future to assess the efficacy of 1882 against drug-resistant strains of enterococci that include vancomycin-resistant enterococcal species. We chose compound 5376 as a lead compound because the MIC value was less than 10 μg/mL against the ATCC *S. aureus* strain.

### 2.2. Ability of Analogues of 5376 to Kill Certain Strains of S. aureus

As a further test, we chose two original compounds, 5376 and 8973, as well as five analogs of the 5376 compound (587084, 5741518, 2082-0182, 2083-1665, and 2083-1773) to perform MIC assays against five well-researched *S. aureus* strains and four other clinical isolates of *S. aureus* (Table 2). Oxacillin and vancomycin—two commercially-available anti-staphylococcal drugs—were used as positive controls, and showed good inhibition of bacterial growth with MIC values of 4 and 1 µg/mL, respectively, for all strains. Compound 5376 showed activity against all *S. aureus* strains that were tested with an MIC_50_ of 4 μg/mL and an MIC_90_ of 8 μg/mL. Minimum bactericial concentration (MBC) assays demonstrated that compound 5376 was bactericidal, reducing viable counts by 2 to 2.5 logs. None of the 5376 analogs had activity against any of the *S. aureus* strains.

Substituted groups in both phenyl X and phenyl Y did not improve the antibacterial activity (Table 3). We found that substituting bromine with a methyl group (compound 5741518) or a hydrogen (compound 5870804) at the same para-position of phenyl X eliminated activity. Other substitutions that eliminated activity were: (1) changing the carboxylate group of phenyl Y to a carboxylate ester (compound 2083-1665) and (2) moving bromine to the meta-position instead of the ortho-position of phenyl X (compound 2083-1773).

### 2.3. Molecular Modeling

**Figure 2:**
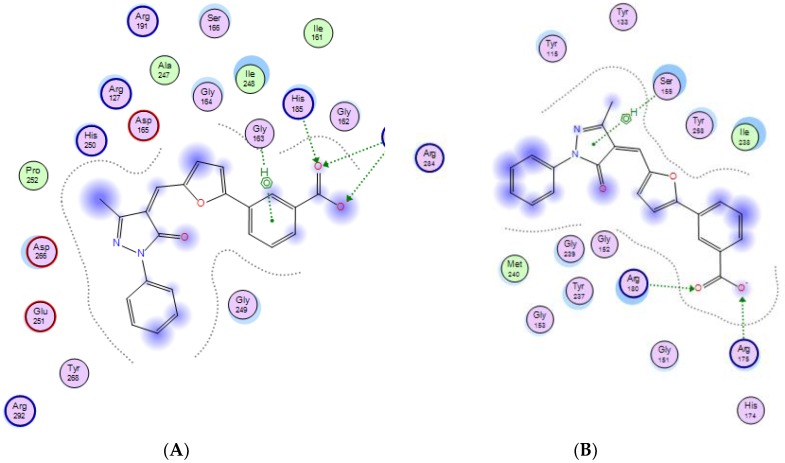
Ligand interaction diagram between compound 5376 and two different thioredoxin reductase crystal structures as generated by Molecular Operating System (MOE). (**A**) Possible active site of *M. tuberculosis* TrxR (PDB file 2A87):5376 complex; (**B**) Possible active site of *S. aureus* TrxR (PDB file 4GCM):5376 complex.

**Figure 3:**
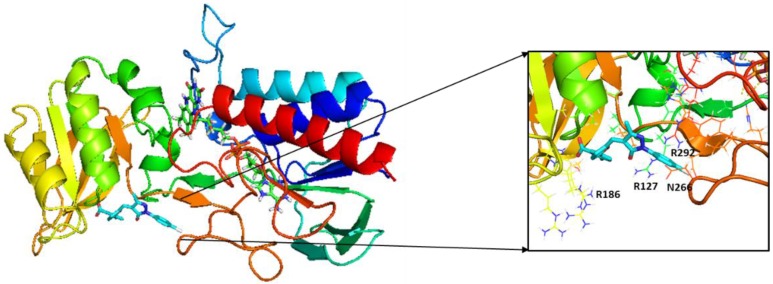

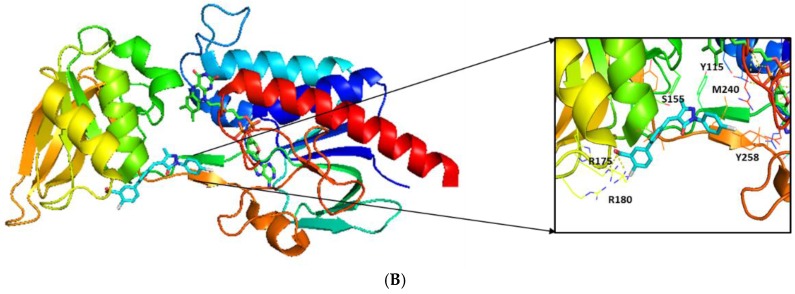
Possible molecular interactions between compound 5376 and *M. tuberculosis* thioredoxin reductase (2A87) and *S. aureus* thioredoxin reductase (4GCM). (**A**) Molecular model of compound 5376 docked in PDB file 4GCM; (**B**) molecular model of compound 5376 docked n PDB file 2A87. TrxR is depicted as ribbons, and compound 5376 is shown along the NADPH site with the co-crystallized FAD adjacent to it. The inset shows the interacting residues within 12 Å of the ligand.

Docking was performed into two different crystal structures: the structure for *S. aureus* TrxR (PDB file 4GCM) and the structure for *M. tuberculosis* TrxR (PDB file 2A87) [7]. Ligand interactions with specific residues of the protein binding site help elucidate the molecular basis of inhibition by our lead compound 5376 with both structures (Figure 2A,B). Based on the molecular model of the compound docked in the *M. tuberculosis* crystal structure (2A87), the guanidinium group of Arg186 forms an ionic bond with the carboxylate end of the compound (Figure 3). Both Arg127 and Arg292 interact with 5376 via their backbone, while Asn266 is a side chain donor. Interestingly, the 5376 compound docked in a slightly different conformation with the *S. aureus* crystal structure (4GCM), predicting interactions with different residues (Figure 3B). In this docking pose, Arg175 and Arg180—two positively-charged arginines—participate in ionic interactions.

### 2.4. Compound 5376 Inhibits M. tuberculosis TrxR Activity and Binding of M. tuberculosis Thioredoxin C (TrxC) to M. tuberculosis TrxR

**Figure 4:**
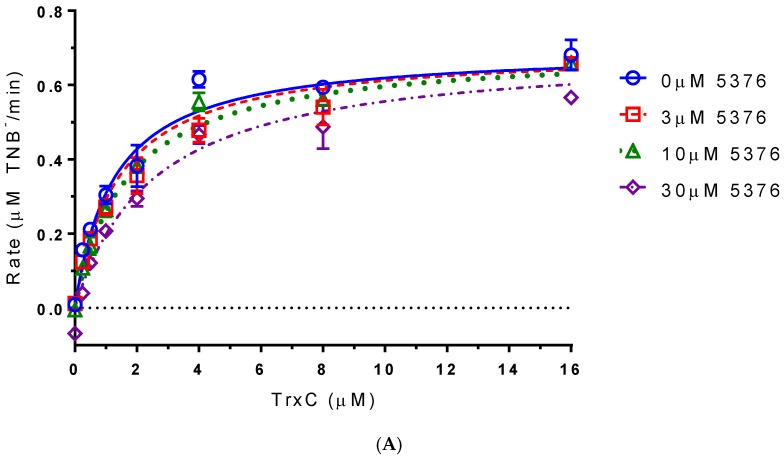

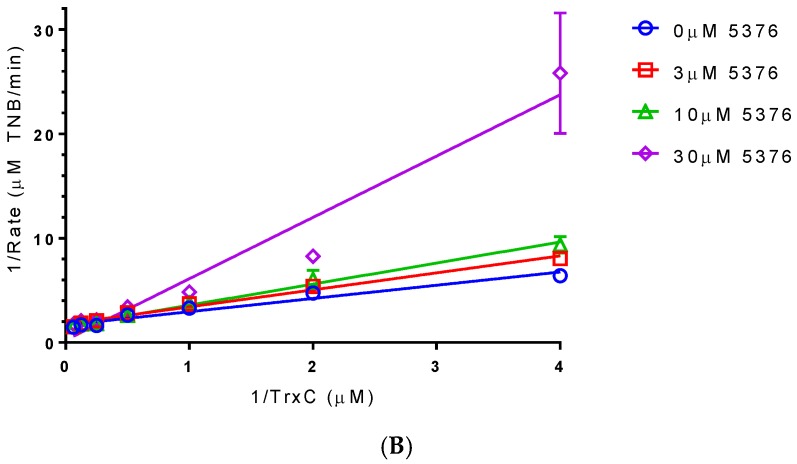
Effect of 5376 on *M. tuberculosis* TrxR-catalyzed DTNB (5,5′-dithiobis(2-nitrobenzoic acid)) reduction by thioredoxin (TrxC). Initial rates measured at indicated TrxC and 5376 concentrations. (**A**) Data were fitted by non-linear regression; (**B**) Data on a Lineweaver–Burk plot with lines fitted by linear regression.

A DTNB (5,5′-dithiobis(2-nitrobenzoic acid)) reduction assay was used to measure the inhibitory capability of compound 5376 in vitro. In this assay, thioredoxin (TrxC) is used as a substrate for TrxR. If a compound binds in place of TrxC, it should prevent the production of TNB (1,3,5-trinitrobenzene) in a dose-dependent manner. To test if compound 5376 bound only the NADPH/TrxC binding site, we examined rates of TrxR-catalyzed NADP reduction at various concentrations of TrxC and 5376. Data fit a competitive inhibition model that assumes that both TrxC and 5376 compete for the same binding site on TrxR (Figure 4A). In this model, 5376 is a competitive inhibitor of TrxC, with a K_i_ (5376) of 1.26 ± 0.11 µM and a K_m_ (TrxC) of 29.7 ± 6.5 µM. Competitive inhibition was also clear when reciprocal data were fit by linear regression on a Lineweaver–Burk plot (Figure 4B).

Interestingly, other research groups have also shown that the Trx system is a novel drug target. A class of benzoisoselenazol compounds were shown to be potent inhibitors of the thioredoxin reductase of *Bacillus anthracis*, the causative agent for anthrax [11]. Biochemical studies found that ebselen and its analogs inhibited the activity of TrxR at a subnanomolar concentration. This study also found that these compounds exhibited a high-barrier against resistance development in *Bacillus subtilis*, *S. aureus*, *Bacillus cereus*, and *M. tuberculosis*. Another study proposed that the mechanism by which ebselen inhibits is by covalent modification of the enzyme’s cysteine residues [12].

In addition, auranofin—an orally bioavailable FDA-approved anti-rheumatic drug—was shown to have potent bactericidal activities against both replicating and non-replicating *M. tuberculosis* based on a cell-based screen for bactericidal compounds [15]. Auranofin was also active against other Gram-positive bacteria, including *B. subtilis*, *E. faecalis*, *E. faecium*, and *S. aureus*. Their biochemical study also showed that auranofin inhibited the bacterial TrxR. They hypothesized that auranofin decreases the reducing capacity of target bacteria, thereby sensitizing them to oxidative stress from the host’s immune system.

The efficacy of auranofin and five of its analogs were further evaluated in vitro against *H. pylori*. Auranofin completely inhibited bacterial growth at 1.2 μM and inhibited the activity purified *H. pylori* TrxR in a cell-free assay (IC50 ~88 nM). However, the compounds were not as toxic against HEK-293T human embryonic kidney cells. Interestingly, they discovered a synergy between auranofin and amoxicillin, suggesting that *H. pylori* infections can be treated by targeting both the reductive enzyme TrxR and cell wall synthesis.

We have identified a new class of antibiotics built on a pyrazolone scaffold that inhibits the growth of drug resistant *S*. *aureus* strains, potentially by inhibiting the *S. aureus* TrxR protein. Future work will try to confirm that the mechanism of action of 5376 is indeed the inhibition of the *S. aureus* TrxR as well as optimize the 5376 lead molecule.

## 3. Experimental Section

### 3.1. Bacterial Species

For the routine MIC screening, a panel of four American Type Culture Collection (ATCC) strains was tested. They included *S. aureus* ATCC 29213, *E. faecalis* ATCC 29212, *E. coli* ATCC 25922, and *P. aeruginosa* ATCC 27853. The following *S. aureus* strains were then used for additional MIC analysis to assess anti-*S. aureus* activity: N315 (USA100), MW2 (USA400), and Newman (MSSA) (provided by Jean Lee (Brigham and Young Hospital, Boston, MA, USA)); JE2 (USA300 provided by the Network on Antimicrobial Resistance in *Staphylococcus aureus* strain repository), and multidrug-resistant MRSA clinical isolates MC7606 (USA300), MC7769 (USA100), MC7827 (USA100), and MC7846 (a vancomycin-intermediate resistance USA100) were supplied by Marshfield Clinic. Strains of *M. smegmatis* and *M. marinum* were obtained from the University of Wisconsin-La Crosse culture collection.

### 3.2. Minimum Inhibitory Concentration (MIC)/Minimum Bactericial Concentration (MBC)

In vitro MIC and MBC determinations were performed on the compounds according to the Clinical and Laboratory Standards Institute (CLSI) guidelines [20] for ATCC strains of *S. aureus*, *E. faecalis*, *E. coli*, and *P. aeruginosa* that were screened. Tetracycline, oxacillin, and vancomycin (Sigma-Aldrich, St. Louis, MO, USA) were included as control antibiotics and correlated with established MIC values for the ATCC strains. All anti-*Mycobacterium* MIC evaluations were performed using MIC assays in Middlebrook 7H9 broth with 10% oleic acid albumin dextrose complex (OADC) as previously described [21]. Isoniazid was used as the positive control for the mycobacterial MICs. All MIC numbers are a compilation of modal values plus range from at least three separate runs.

### 3.3. Thioredoxin Reductase-DTNB Assay

The activity of *M. tuberculosis* TrxR was examined using a DTNB (5,5′-dithiobis(2-nitrobenzoic acid)) reduction assay [22]. TrxR and TrxC were expressed and purified as previously described [23]. In the assay, TrxR used NADPH to oxidize TrxC, which spontaneously reduced DTNB. The production of TNB (1,3,5-trinitrobenzene) was monitored by measuring its absorbance at 412 nm in a SpectraMax M5 multi-mode plate reader (Molecular Devices, Sunnyvale, CA, USA). The assay was used to examine the inhibition kinetics of 5376. Each 200 µL assay contained 200 mM KPO_4_ pH 7.2 mM 

**Figure A1:**
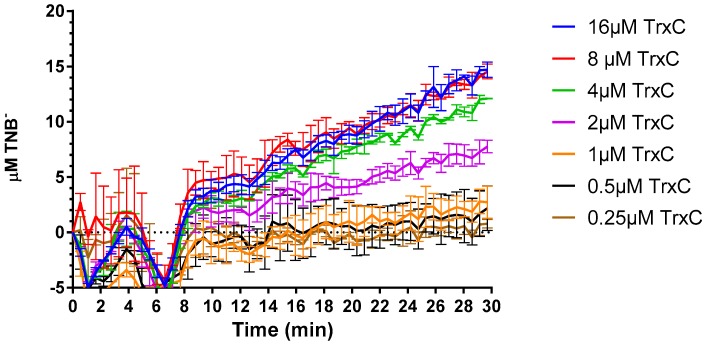
Reaction progress curves of wells containing varying concentrations of TrxC and 30 μM 5376. The rate of the reaction was calculated after 8.8 minutes of data collection, which is where progress curves become linear.

EDTA, 2 µM bovine serum albumin, 1 mM DTNB, 500 µM NADPH, 10 nM TrxR, and 1% dimethyl sulfoxide. Concentrations of CSD^3^ 5376 tested were 30, 10, 3, and 0 µM, and concentrations of TrxC tested were a seven-point 1:2 dilution series ranging between 16 µM and 0.25 µM, in duplicate Assays were initiated by the addition of TrxR. Assays were done in clear 96-well non-treated microplates (Greiner BioOne 655101, Monroe, LA, USA). Since 5376 has color, compound background absorbance control wells were included that lacked TrxR and TrxC, but included all other assay components. Reaction progress was monitored for 30 min. The compound background absorbance for each concentration was subtracted from experimental wells for each time point before calculating the rate of reaction. The absorbance values were converted to concentration of TNB produced using the extinction coefficient 14,150 M^−1^·cm^−1^. The rate of reaction was calculated after 8.25 min to assure a linear rate (see Appendix A Figure A1). The data were fitted to a competitive inhibition model using Graphpad Prism 6.04.

### 3.4. Docking

The structures of the TrxR proteins in this study were obtained from the protein data bank (www.pdb.org) [24]. The accession code for the *M. tuberculosis* thioredoxin reductase is 2A87, and the accession code for the *S. aureus* subsp. *aureus* Mu50 strain is 4GCM. Compounds were docked into PDB files 2A87 and 4GCM that were stripped of their ligands, water molecules, and counterions. We have decided to dock with FAD included in the structures, since both crystal structures have FAD co-crystallized. Using UCSF Chimera′s Dock Prep module, histidine protonation states were calculated, and incomplete side chains were automatically filled [25]. Three-dimensional conformations of the compounds were generated using Open Babel, and were positioned using a rigid receptor and flexible ligand orienting code in AutoDock 4.2.1 (AD4, Scripps Research Institute, La Jolla, CA, USA) [26]. Both receptor and ligand were seeded with Gasteiger charges. AD4 was used for all dockings in this study. In general, the docking parameters were kept to their default values. However, the number of independent genetic algorithm runs was increased from 10 to 50, and was processed using the built-in clustering analysis with a 2.0 Å cutoff [27].

## 4. Conclusions

The in silico docking analysis against the *M. tuberculosis* TrxR identified in vitro enzyme inhibitors, but failed to generate a viable lead compound against *M. tuberculosis* due to negative MIC results; however, our results have identified and may encourage the development of more potent and specific analogs of a pyrazolone-based lead molecule 5376 as possible *S. aureus* antibacterial agents. The pyrazolone scaffold of lead compound 5376 showed a positive MIC result that was sufficient to inhibit several strains of *S. aureus*, including multidrug resistant clinical isolates. Although the 5376 compound did not have the same predicted affinity for the *S. aureus* TrxR active site as compared to the *M. tuberculosis* TrxR, it was still a close fit and suggests but does not prove that compound 5376 targets the *S. aureus* TrxR. Future studies will focus on confirming that the mechanism of action involves inhibiting the *S. aureus* TrxR enzyme and optimizing the pyrazolone compound to increase efficacy and in vivo properties such as pharmacokinetics.

## Figures and Tables

**Table 1:** The minimum inhibitory concentration (MIC) results for the compounds tested against a panel of six bacterial species.

MIC (μg/mL)						
Compound	*S. aureus*	*E. faecalis*	*E. coli*	*P. aeruginosa*	*M. smegmatis*	*M. marinum*
8973	64 (64) ^a^	>128 (>128)	>128 (>128)	>128 (>128)	>128 (>128)	>128 (>128)
6702	64 (64)	>128 (>128)	>128 (>128)	>128 (>128)	>128 (>128)	>128 (>128)
1108	>128 (>128)	>128 (>128)	>128 (>128)	>128 (>128)	>128 (>128)	>128 (>128)
2628	>128 (>128)	>128 (>128)	>128 (>128)	>128 (>128)	>128 (>128)	>128 (>128)
9010	>128 (>128)	>128 (>128)	>128 (>128)	>128 (>128)	128 (128)	>128 (>128)
1632	>128 (>128)	>128 (>128)	>128 (>128)	>128 (>128)	>128 (>128)	>128 (>128)
5376	8 (4-8)	128 (128)	>128 (>128)	>128 (>128)	128 (128)	>128 (>128)
1882	32 (32)	32 (32)	>128 (>128)	>128 (>128)	128 (128)	>128 (>128)
3719	64 (64)	128 (128)	>128 (>128)	>128 (>128)	>128 (>128)	>128 (>128)
Tetracycline	0.5 (0.25–0.5)	32 (32)	2 (1–2)	16 (16)	ND	ND
Isoniazid	ND ^b^	ND	ND	ND	4	8

^a^ The MICs represent the modal values (range) from at least three separate runs; ^b^ ND = Not determined. *E. coli*: *Escherichia coli*; *E. faecalis*: *Enterococcus faecalis*; *M. marinum*: *Mycobacterium marinum. M. smegmatis*: *Mycobacterium smegmatis*; *P. aeruginosa*: *Pseudomonas aeruginosa*; *S. aureus*: *Staphylococcus aureus*.

**Table 2:** MIC results for select compounds tested against nine strains of *Staphylococcus aureus*.

MIC (μg/mL)									
Compound	ATCC ^a^	MW2	JE2	Newman	N315	MC7606 ^b^	MC7769	MC7827	MC7846
8973	>128 ^c^ (>128)	>128 (>128)	>128 (>128)	>128 (>128)	>128 (>128)	>128 (>128)	>128 (>128)	>128 (>128)	>128 (>128)
5376	8 (4-8)	4 (4)	8 (8)	4 (4)	4 (2–4)	2 (2)	4 (4–8)	8 (8)	4 (4)
5741518	>128 (>128)	>128 (>128)	>128 (>128)	>128 (>128)	>128 (>128)	>128 (>128)	>128 (>128)	>128 (>128)	>128 (>128)
5870804	16 (16)	32 (32)	32 (32)	64 (64)	32 (32)	4 (4)	64 (64)	32 (32–64)	16 (16)
2082-0182	>128 (>128)	>128 (>128)	>128 (>128)	>128 (>128)	>128 (>128)	>128 (>128)	>128 (>128)	>128 (>128)	>128 (>128)
2083-1665	>128 (>128)	>128 (>128)	>128 (>128)	>128 (>128)	>128 (>128)	>128 (>128)	>128 (>128)	>128 (>128)	>128 (>128)
2083-1773	>128 (>128)	>128 (>128)	>128 (>128)	>128 (>128)	>128 (>128)	>128 (>128)	>128 (>128)	>128 (>128)	>128 (>128)
Tetracycline	0.25 (0.25–0.5)	0.25 (0.25)	0.25 (0.25)	0.25 (0.25)	0.25 (0.25)	32 (16–32)	0.25 (0.25)	32 (32)	0.125 (0.125)
Oxacillin	0.25 (0.25)	8 (8–16)	8 (8–6)	0.5 (0.5)	16 (16)	4 (4)	4 (4)	4 (4)	4 (4–8)
Vancomycin	0.5 (0.5)	1 (1)	1 (1)	1 (1)	1 (1)	1 (1)	1 (1)	1 (1)	4 (4)

^a^ ATCC is the American Type Culture Collection strain 29213; ^b^ Marshfield Clinic *S. aureus* clinical isolate; ^c^ The MICs represent the modal values (range) from at least three separate runs.

**Table 3:** Structure–activity relationship of lead compound 5376 and its derivatives targeting drug-resistant *Staphylococcus aureus*.

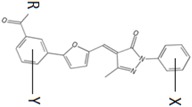								
Compound	R	X	Y	MIC (µg/mL) MC7606	MIC (µg/mL) MC7769	MIC (µg/mL) MC7827	MIC (µg/mL) MC7846	Docking Energy (kcal/mol)
5376	O	4-Br	4-Cl	2	4	8	4	−9.3
5741518	O	4-CH_3_	4-Cl	>128	>128	>128	>128	−8.7
5870804	O	4-Br	H	4	64	32	16	−10.9
2082-0182	O	H	4-Cl	>128	>128	>128	>128	−9.0
2083-1665	OCH_3_	4-Br	4-Cl	>128	>128	>128	>128	−8.6
2083-1773	O	3-Br	4-Cl	>128	>128	>128	>128	−9.8

## Abbreviations

The following abbreviations are used in this manuscript:

DTNB	Ellman′s Reagent (5,5-dithio-bis-(2-nitrobenzoic acid))
DTT	1,4-Dithiothreitol
TrxR	Thioredoxin Reductase
Trx	Thioredoxin
PDB	Protein Data Bank
